# Public perceptions of the effectiveness of recommended non-pharmaceutical intervention behaviors to mitigate the spread of SARS-CoV-2

**DOI:** 10.1371/journal.pone.0241662

**Published:** 2020-11-04

**Authors:** Monica L. Kasting, Katharine J. Head, Jane A. Hartsock, Lynne Sturm, Gregory D. Zimet

**Affiliations:** 1 Department of Public Health, Purdue University, West Lafayette, Indiana, United States of America; 2 Cancer Prevention and Control Program, Indiana University Simon Comprehensive Cancer Center, Indianapolis, Indiana, United States of America; 3 Department of Communication Studies, Indiana University-Purdue University Indianapolis, Indianapolis, Indiana, United States of America; 4 Department of Clinical and Organizational Ethics, Indiana University Health, Indianapolis, Indiana, United States of America; 5 Center for Bioethics, Indiana University, Indianapolis, Indiana, United States of America; 6 Medical Humanities & Health Studies Program, Indiana University-Purdue University Indianapolis, Indianapolis, Indiana, United States of America; 7 Department of Pediatrics, Indiana University School of Medicine, Indianapolis, Indiana, United States of America; Drexel University School of Public Health, UNITED STATES

## Abstract

**Background:**

The COVID-19 pandemic is an unprecedented public health threat, both in scope and response. With no vaccine available, the public is advised to practice non-pharmaceutical interventions (NPI) including social distancing, mask-wearing, and washing hands. However, little is known about public perceptions of the effectiveness of these measures, and high perceived effectiveness is likely to be critical in order to achieve widespread adoption of NPI.

**Methods:**

In May 2020, we conducted a cross-sectional survey among U.S. adults (N = 3,474). The primary outcome was a six-item measure assessing perceived effectiveness of recommended behaviors to prevent SARS-CoV-2 infection from 1 (not at all effective) to 5 (extremely effective). The sample was divided into “higher” and “lower” perceived effectiveness groups. Covariates included demographics, healthcare characteristics, and health beliefs. Variables that were significant at p<0.01 in bivariate analyses were entered into a multivariable logistic regression and a best-fit model was created using a cutoff of p<0.01 to stay in the model.

**Results:**

Mean age was 45.5 years and most participants were non-Hispanic White (63%) and female (52.4%). The high perceived effectiveness group was slightly larger than the low perceived effectiveness group (52.7% vs. 47.3%). Almost all health belief variables were significant in the best-fit regression model. COVID-19-related worry (aOR = 1.82; 95% CI = 1.64–2.02), and perceived threat to physical health (aOR = 1.32; 95% CI = 1.20–1.45) were positively associated with perceived effectiveness while perceived severity of COVID-19 (0.84; 95% CI = 0.73–0.96) and perceived likelihood of infection (0.85; 95% CI = 0.77–0.94) switched directions in the adjusted model and were negatively associated with perceived effectiveness.

**Conclusions:**

This research indicates people generally believe NPI are effective, but there was variability based on health beliefs and there are mixed rates of engagement in these behaviors. Public health efforts should focus on increasing perceived severity and threat of SARS-CoV-2-related disease, while promoting NPI as effective in reducing threat.

## Introduction

In late December 2019, Chinese authorities first notified the World Health Organization (WHO) of a cluster of cases of pneumonia in the Chinese Wuhan Hubei Province (1). These pneumonia cases were determined to be caused by a novel coronavirus named severe acute respiratory syndrome coronavirus 2 (SARS-CoV-2), which is now known to cause the new coronavirus disease (COVID-19) [1,2]. Within the first few months of the 2020 calendar year, the virus spread rapidly around the world and, particularly, in the U.S. On March 11, 2020, WHO officially declared COVID-19 a pandemic [1]. By mid-July 2020, the U.S. had over 3 million cases of COVID-19 and almost 140,000 deaths [3]. At the time of this research (May 2020), there were no pharmaceutical options for prevention or cure. Therefore, to curb the spread of the virus, communities were asked to enact non-pharmaceutical interventions (NPI) to prevent infections until a vaccine is developed and protection through herd immunity can be achieved. Because SARS-CoV-2 is thought to be spread mainly through person-to-person transmission, implementation of personal protective practices have been recommended to slow viral spread [4]. Specifically, the U.S. Centers for Disease Control and Prevention (CDC) suggests several mitigation strategies including (but not limited to): 1. frequent handwashing, 2. avoiding close contact with others (social distancing), 3. wearing masks, 4. covering coughs and sneezes, 5. cleaning and disinfecting surfaces, and 6. being alert for COVID-19 symptoms [5].

There has been inconsistent adoption of mitigation strategies in the U.S., despite the fact that COVID-19 cases are increasing in large parts of the country [3]. This problem has been exacerbated by inconsistent and, at times, contradictory messaging, particularly at the federal level [6]. Specifically, in the early stages of the pandemic, guidance from public health organizations was that wearing masks, in particular, was not necessary if the person was not sick [7]. However, at that point in time, there were personal protective equipment shortages in hospitals and health officials were also attempting to prioritize mask supplies for health professionals [7]. Furthermore, the WHO did not issue a recommendation for the general public to wear masks until June, 2020 [8]. Prior to that, they had issued a statement in April 2020 stating that mask use was not supported by the evidence [9]. More recently, there is increasing evidence of the effectiveness of mask-wearing to prevent SARS-CoV-2 spread [10] and masks are now recommended for use in public by the CDC and mandated in 20 states across the U.S. [5,11]. Nevertheless, some public figures and elected officials have refused to be photographed wearing masks while out in public [12], despite evidence of the effectiveness of mask-wearing for decreasing the spread of SARS-CoV-2 across populations [13]. Implementing effective mitigation strategies has been further complicated by officials publicly down-playing the risk posed by the virus [14] and stating that it will resolve on its own [15], while privately acknowledging the severity of the disease [16]. Recent polling has indicated only one-third of Americans always wear a mask when they are in public [17]. And while a large proportion of the population (84%) reported in late March, 2020 that they practiced social distancing, this proportion decreased by 10% by the end of April, despite a rise in cases and deaths [3,18]. Additional research has found, in general, U.S. residents had lower public approval of NPI measures such as those promoted by the CDC, compared to their counterparts in areas of Asia and Central and South America [19]. Furthermore, a systematic review found individuals were ambivalent about adopting personal distancing behavior due to concerns about social stigma [20].

Previous research has shown distrust of the government hinders cooperation with public health recommendations during a public health crisis, such as the current pandemic [21]. Furthermore, the rapidly emerging new information and naturally evolving science in the face of a novel virus may lead the public to distrust information from public health organizations, if it seems to change frequently [22]. Given some of the information related to these NPIs has changed as the scientific information has evolved, mistrust of information could decrease the perceived effectiveness of mitigation strategies recommended by the CDC, and may therefore lead to decreased engagement in practices that slow the spread of the virus. Moreover, previous studies using simulated infectious disease outbreaks found that an individual’s intention to engage in social distancing behaviors increased if the person believed their behaviors would reduce the threat of the disease [23]. Theoretical models like the Health Belief Model (HBM) and Extended Parallel Process Model suggest that cues to action, perceptions of severity and susceptibility of the threat, and perceived response efficacy (e.g., belief that the recommended behavior will mitigate a threat) are important predictors of behavior uptake [24,25]. However, little is known about how the public perceives NPIs within the context of the current pandemic. Therefore, this study aimed to: 1) understand perceived effectiveness of CDC recommended NPIs to mitigate the spread of SARS-CoV-2 and 2) examine factors associated with perceived effectiveness to identify potential targets for future intervention efforts.

## Methods

### Participants and recruitment

An online Qualtrics survey was conducted the week of May 4^th^-May 11^th^, 2020. Eligible participants were recruited via e-mail invitation by Dynata, a survey research company that maintains panels of volunteer survey respondents who receive monetary incentives for participation. Dynata employs recruitment quotas based on U.S census data to ensure the sample is balanced by age, gender, race/ethnicity, and geographic location. Eligibility criteria included being 18 years or older and able to read English. Participants on the Dynata survey panel are compensated in points based on study length. The study was given exempt status by the Indiana University Institutional Review Board.

### Measures

While this study examined perceptions of SARS-CoV-2 infection and spread, pretesting of the survey instruments determined the term “COVID-19” was more appropriate for lay audiences, because SARS-CoV-2 is used in lay communication less frequently. Therefore, we used the term “COVID-19” exclusively in the survey items, even if we were talking about the virus itself.

#### Perceptions of non-pharmaceutical interventions to flatten the curve of COVID-19 disease

A six-item measure was used to assess participants’ perceptions of the effectiveness of NPIs to prevent SARS-CoV-2 infection and spread. Each item involved a behavior the participant themselves could practice and was measured on a five-point Likert scale from 1 (not at all effective) to 5 (extremely effective). The items, exactly as they appeared in the survey, can be found in S1 Appendix. Three of the six items measured the perceived effectiveness of preventing yourself from catching COVID-19 and included: 1) practicing social distancing by leaving at least six feet between you and other people (this does not include people you live with), 2) frequently washing your hands for 20 seconds with warm water and soap, and 3) avoiding touching your face. Three of the six items measured the perceived effectiveness of preventing yourself from spreading COVID-19 to others and included: 1) wearing a mask anytime you leave the house to go out in public, 2) practicing social distancing by leaving at least six feet between you and other people (this does not include people you live with), and 3) covering your mouth when you cough. The six items created a reliable perceived effectiveness scale with a Cronbach’s alpha of 0.91. We conducted an exploratory factor analysis, which extracted a single factor that accounted for 69% of the variance of the data, indicating these six items reflect a single construct. We then created a mean effectiveness score so that each person had a score on a scale of 1–5. For purposes of analyses, we divided the sample using a median split, creating two groups: those who had lower perceived effectiveness of the NPI measures (score range: 1–3.99; 47.3% of the sample) and those who had higher perceived effectiveness (score range: 4.00–5.00; 52.7% of the sample).

### Covariates

Covariates fell into three categories: demographic characteristics, healthcare characteristics, and health belief variables. Healthcare characteristics included having a health condition that would make COVID-19 more severe, believing they have had COVID-19, ever having received a SARS-CoV-2 test (and the result of the test, among those who said “yes”), and personally knowing anyone who had COVID-19 disease.

Health belief variables included:

#### Altruism

Participants were asked a modified version of a previously validated 18-item altruism scale [26]. This original scale consisted of 18 questions assessing frequency of engagement in various altruistic activities (e.g. helping a stranger push their car out of the snow or mud) on a Likert-type scale from 1 = never to 5 = very often. We conducted a principal components exploratory factor analysis, which extracted two factors. We labeled the first factor, which consisted of five items (Cronbach’s α = 0.83), *high commitment altruism* (i.e., behaviors that require a relatively high level of personal involvement; e.g., “I have offered my seat to a stranger who was standing”). We labeled the second factor, which consisted of four items (Cronbach’s α = 0.81), *low commitment altruism* (i.e., behaviors that require a relatively low level of personal involvement; e.g., “I have given money to charity”). We calculated a mean score for each of the altruism subscales.

Perceived severity of COVID-19 disease was measured using a four-item scale adapted from a measure of perceived Ebola severity [27]. Items assessed participants’ perceptions of the severity of COVID-19 disease (e.g. “I am afraid that I may die if I contract COVID-19”) on a scale of 1 (strongly disagree) to 5 (strongly agree) so that higher scores indicate higher perceived severity. The four items (Cronbach’s α = 0.71) were summed and averaged to derive a single perceived severity score. The perceived severity questions were only asked of the participants who indicated they did not believe they had previously had COVID-19 disease.

COVID-19-related worry was assessed with a three-item scale modified from the literature [28,29]. Items assessed participants’ worry related to getting COVID-19 (e.g. “The possibility of getting infected in the future with COVID-19 concerns me”). Participants responded to each item on a 5-point Likert scale from 1 = strongly disagree to 5 = strongly agree. The three items (Cronbach’s α = 0.82) were summed and averaged into a single scale with higher numbers indicating higher COVID-19-related worry. As with the perceived severity variable, the COVID-19-related worry questions were also only asked of the participants who indicated they did not believe they had previously had COVID-19 disease.

Perceived personal threat of COVID-19 disease was analyzed with two separate items: perceived likelihood of infection and perceived threat to physical health. Perceived likelihood of infection was measured by asking participants “How likely do you believe it is that you will get infected with COVID-19?” Participants responded on a 5-point Likert-type scale where 1 = not at all to 5 = extremely. Perceived threat to physical health was measured by asking participants “If you got infected with COVID-19, how threatening would it be to your physical health?” Participants responded on a 5-point Likert-type scale where 1 = not at all to 5 = extremely.

Perceived community threat was assessed with a single item, “Do you think COVID-19 infection is a major problem in your community?” with a binary yes/no response option.

### Analysis

First, we described the study sample using n (%) or means and standard deviations. We then compared the “lower perceived effectiveness” and “higher perceived effectiveness” groups using chi-square or t-tests, as appropriate. Any variable that was significant at *p*<0.01 in bivariate comparisons was included in an adjusted logistic regression model with the binary lower/higher perceived effectiveness of COVID-19 prevention measures as the outcome. We then used a backward selection process to create a reduced model with *p*<0.01 needed to stay in the model. Because participants who believed they previously had COVID-19 were not asked perceived severity and perceived susceptibility questions, those participants were excluded from the logistic regression analyses so we could understand perceived effectiveness of COVID-19 prevention measures, while accounting for perceived severity and susceptibility, among those not previously thought to be infected.

## Results

A total of 16,706 invitations were sent out for the survey, 4,042 people opened the survey, 351 indicated they did not wish to participate, and 42 indicated they were younger than 18 years. A total of 3,586 completed the survey and 3,474 answered all of the questions regarding the effectiveness of the recommended prevention measures and were included in the statistical analyses comparing the groups. The mean age of the sample was 45.5 (SD = 16.9); the sample was 47.3% male, 52.4% female, and 0.3% other. The majority of participants identified as non-Hispanic White (62.8%), with other groups represented, including non-Hispanic Black/African American (15.3%), and Hispanic (14.0%). Participants were fairly evenly split by political ideology with 1,069 (31.9%) indicating their political views were liberal, 1,280 (38.2%) were moderate, and 1,005 (30.0%) were conservative. Approximately one-tenth of participants (n = 360; 10.1%) indicated they believed they had previously had COVID-19 disease. More than one-third personally knew someone who had COVID-19, i.e., the person either had a positive SARS-CoV-2 test (n = 881; 24.9%) or they had symptoms, but were unable to get tested (n = 354; 10.0%). On average, perceived worry (M = 3.46; SD = 1.08) was higher than perceived severity of disease (M = 3.02; SD = 0.87) (*p*<0.0001) and most people thought COVID-19 was a major problem in their community (n = 2037; 57.8%). For additional sample description, see Table 1.

**Table 1:** Sample description by lower vs. higher perceived effectiveness of CDC recommended behaviors to prevent the spread of COVID-19.

Variable	Total Sample n(%) or mean(SD) n = 3474	Lower perceived effectiveness n = 1642; 47.3%	Higher perceived effectiveness n = 1832; 52.7%	p-value for difference
*Demographic Characteristics*				
Age	45.7 (16.9)	42.88 (16.9)	48.22 (16.6)	**<0.0001**
Geographic Region				0.052
*Northeast*	708 (20.4)	314 (19.4)	394 (21.6)	
*Southeast*	899 (25.9)	406 (25.1)	493 (27.0)	
*Midwest*	744 (21.4)	383 (23.6)	361 (19.8)	
*Southwest*	374 (10.8)	180 (11.1)	194 (10.6)	
*West*	718 (20.7)	337 (20.8)	381 (20.9)	
*Missing*	31 (0.9)			
Sex				**<0.0001**
*Male*	1638 (47.2)	834 (51.2)	804 (44.1)	
*Female*	1813 (52.2)	795 (48.8)	1018 (55.9)	
*Missing*	23 (0.7)			
Race/Ethnicity				0.450
*Non-Hispanic White*	2176 (62.6)	1010 (62.4)	1166 (64.2)	
*Non-Hispanic Black/African American*	514 (14.8)	259 (16.0)	255 (14.0)	
*Non-Hispanic Other*^a^	265 (7.6)	125 (7.7)	140 (7.7)	
*Hispanic*	479 (13.8)	225 (13.9)	254 (14.0)	
*Missing*	40 (1.2)			
Relationship status				0.024
*Partnered*	2012 (57.9)	916 (56.3)	1096 (60.1)	
*Not partnered*	1437 (41.4)	710 (43.7)	727 (39.9)	
*Missing*	25 (0.7)			
Children living in home				0.129
*No*	2427 (69.9)	1115 (70.6)	1312 (73.0)	
*Yes*	950 (27.3)	464 (29.4)	486 (27.0)	
*Missing*	97 (2.8)			
Education				**0.002**
*Less than HS grad*, *HS grad*, *GED*	786 (22.6)	417 (25.9)	369 (20.3)	
*Some college/Associate’s degree*	979 (28.2)	442 (27.4)	537 (29.6)	
*Bachelor’s degree*	1009 (29.0)	464 (28.8)	545 (30.0)	
*Graduate school*	653 (18.8)	290 (18.0)	363 (20.0)	
*Missing*	47 (1.4)			
Work in healthcare				**<0.0001**
*Yes*, *currently*	522 (15.0)	299 (18.8)	223 (12.4)	
*Yes*, *in the past*	503 (14.5)	257 (16.2)	246 (13.7)	
*No*, *never*	2358 (67.9)	1032 (65.0)	1326 (73.9)	
*Missing*	91 (2.6)			
Currently employed (earning income)				**0.001**
*Yes*, *full time (35+ hours per week)*	1158 (33.3)	550 (34.0)	608 (33.4)	
*Yes*, *part time*	487 (14.0)	262 (16.2)	225 (12.4)	
*Yes*, *furloughed with pay*	91 (2.6)	56 (3.5)	35 (1.9)	
*Yes*, *furloughed without pay*	192 (5.5)	89 (5.5)	103 (5.7)	
*No*, *looking for work*	364 (10.5)	160 (9.9)	204 (11.2)	
*No*, *not looking for work*^b^	1107 (31.9)	485 (30.0)	622 (34.2)	
*Other*	36 (1.0)	14 (0.9)	22 (1.2)	
*Missing*	39 (1.1)			
Household income (2019)				**0.003**
*Less than $25*,*000*	1044 (30.1)	543 (34.0)	501 (28.1)	
*$25*,*000-$74*,*999*	1040 (29.9)	476 (29.8)	564 (31.6)	
*$75*,*000-$149*,*999*	944 (27.2)	421 (26.3)	523 (29.3)	
*$150*,*000 or more*	354 (10.2)	158 (9.9)	196 (11.0)	
*Missing*	92 (2.6)			
Political views				**<0.0001**
*Liberal*	1032 (29.7)	407 (26.9)	625 (35.8)	
*Moderate*	1244 (35.8)	580 (38.3)	664 (38.0)	
*Conservative*	983 (28.3)	526 (34.8)	457 (26.2)	
*Missing*	215 (6.2)			
*Healthcare Characteristics*				
Pre-existing condition that would make COVID-19 more severe				**<0.0001**
*Yes*	1202 (34.6)	509 (31.3)	693 (38.1)	
*No*	2242 (64.5)	1117 (68.7)	1125 (61.9)	
*Missing*	30 (0.9)			
Believe they’ve been infected with COVID-19				**0.004**
*Yes*	341 (9.8)	174 (10.6)	167 (9.1)	
*Not sure*	581 (16.7)	304 (18.6)	277 (15.1)	
*No*	2543 (73.2)	1157 (70.8)	1386 (75.7)	
*Missing*	9 (0.3)			
Ever received a COVID-19 test				**<0.0001**
*Yes*	389 (11.2)	226 (14.0)	163 (9.0)	
Result of test				
Positive: 109 (3.1)		*50 (22*.*6)*	*59 (36*.*2)*	**<0.0001**
Negative: 234 (6.7)		*137 (62*.*0)*	*97 (59*.*5)*	
Still waiting on results: 41 (1.2)		*34 (15*.*4)*	*7 (4*.*3)*	
*No*	3035 (87.4)	1386 (86.0)	1649 (91.0)	
*Missing*	50 (1.4)			
Know anyone who has had COVID-19				**<0.0001**
*Yes*, *they had positive test*	855 (24.6)	345 (21.3)	510 (28.1)	
*Believe so but unable to get tested*	343 (9.9)	208 (12.8)	135 (7.4)	
*No*, *didn’t know anyone with COVID-19*	2240 (64.5)	1070 (65.9)	1170 (64.5)	
*Missing*	36 (1.0)			
*Health Belief Variables*				
Total Altruism Scale	2.83 (0.79)	2.73 (0.72)	2.92 (0.84)	**<0.0001**
High Commitment Altruism	2.53 (0.95)	2.48 (0.85)	2.58 (1.02)	**0.001**
Low Commitment Altruism	3.39 (0.91)	3.18 (0.87)	3.58 (0.90)	**<0.0001**
Mean perceived severity of COVID-19 (4-items, range:1–5)	3.02 (0.88)	2.84 (0.83)	3.17 (0.89)	**<0.0001**
Mean COVID-19-related worry (3-items; range:1–5)	3.47 (1.08)	3.07 (1.05)	3.81 (0.98)	**<0.0001**
Threat of COVID-19 infection (individual items, of those not previously infected)				
*Likelihood of infection (1 = not at all; 5 = extremely)*	2.32 (1.03)	2.20 (0.98)	2.44 (1.05)	**<0.0001**
*Threat to physical health (1 = not at all; 5 = extremely)*	3.06 (1.23)	2.74 (1.18)	3.34 (1.20)	**<0.0001**
Believe COVID-19 is major problem in community				**<0.0001**
*Yes*	1971 (56.7)	781 (48.5)	1190 (65.9)	
*No*	1444 (41.6)	829 (51.5)	615 (34.1)	
*Missing*	59 (1.7)			

^a^Other race ethnicity = American Indian or Native American, Asian, Native Hawaiian or other Pacific Islander, Other, Prefer not to answer.

^b^“No, not looking for work” includes retired, students, disabled, stay-at-home parent, and homemaker.

**Fig 1:**
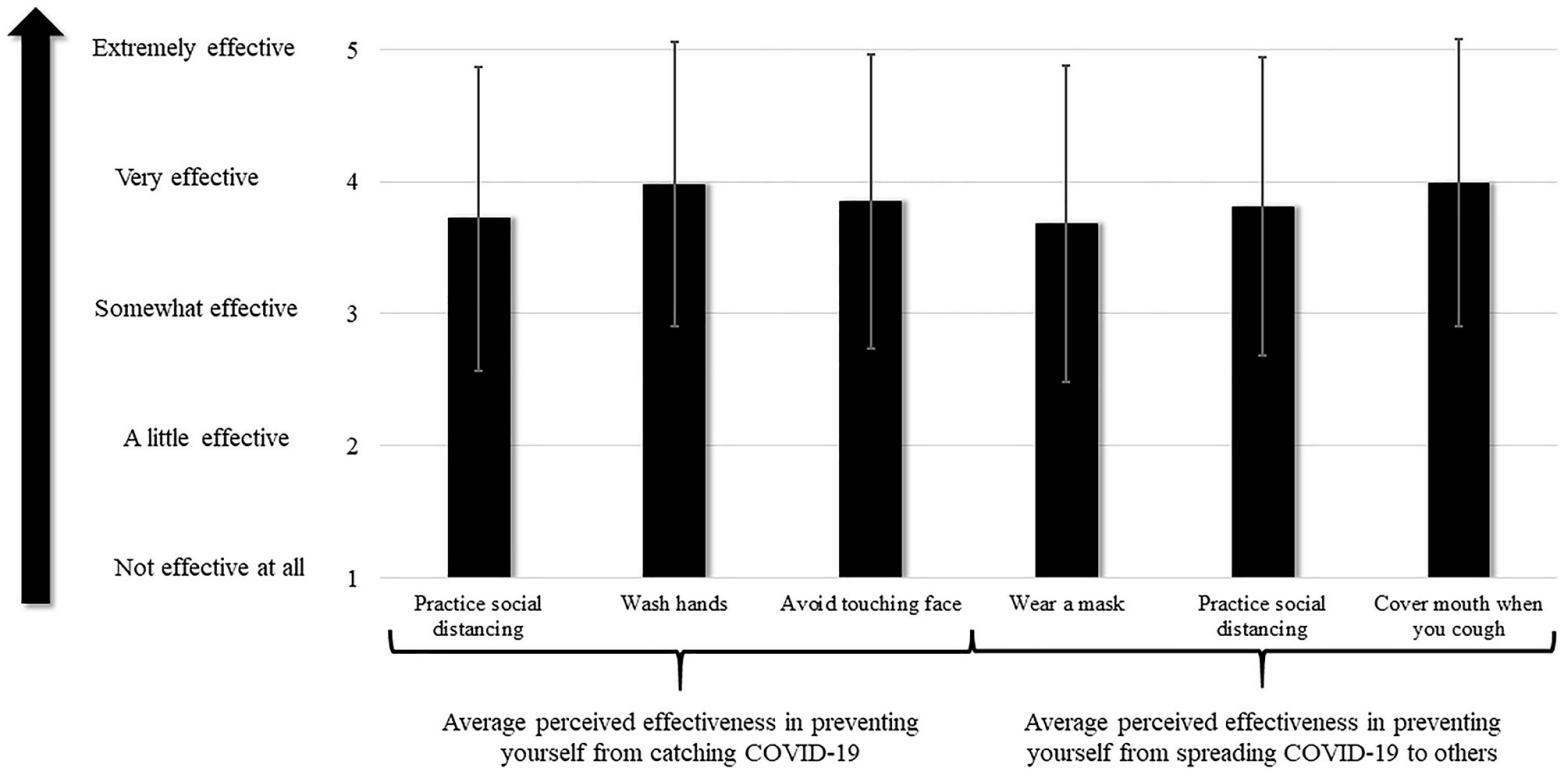
Average perceived effectiveness of non-pharmaceutical COVID-19 prevention behaviors. *Error bar indicate standard deviation.

Overall, perceived effectiveness of the six CDC recommended items was high. The lowest individual mean of perceived effectiveness value was for wearing a mask to prevent spreading the virus to others (m = 3.68; SD = 1.2) and the highest individual mean was for covering your mouth when you cough to prevent spreading the virus to others (m = 3.99; SD = 1.09). See Fig 1 for a graphical representation of the mean scores for all six items.

### Bivariate comparisons

There were differences between the lower and higher perceived effectiveness groups on every healthcare and health belief variable (Table 1). Notably, the lower perceived effectiveness group had lower perceived severity of COVID-19 (2.84 vs. 3.17; *p*<0.0001), lower COVID-19-related worry (3.07 vs. 3.81; *p*<0.0001), lower perceived likelihood of infection (2.20 vs. 2.44; *p*<0.0001), and lower scores on both altruism scales. A larger percentage of the higher perceived effectiveness group reported they did not believe they had been infected with COVID-19 (75.7% vs. 70.8%; *p* = 0.004). While this was significant, as noted above, it was not included in the regression model due to the fact that only those who answered “no” were asked questions regarding severity and worry. However, whether they had received a test to check for COVID-19 was asked of everyone and included in the regression model. A larger percentage of the lower perceived effectiveness group had ever received a test to check for COVID-19 (14% vs 9% in the higher perceived effectiveness group; *p*<0.0001) but, of the ones that were tested, they had a lower positivity rate (22.6% vs. 36.2%; *p*<0.0001).

There were some demographic characteristics that were not significantly different between groups, including geographic region (p = 0.052), race/ethnicity (p = 0.450), and whether there were children living in the home (p = 0.129). These variables, along with relationship status (p = 0.024) were not significant at *p*<0.01 and were not included in the logistic regression models.

Of the variables that were significant at *p*<0.01, we conducted further bivariate analyses to determine the odds of being in the higher perceived effectiveness group. These results can be found in Table 2. Specifically, for the health belief variables, high commitment altruism (OR = 1.12; 95% CI = 1.04–1.20), low commitment altruism (OR = 1.67; 95% CI = 1.54–1.80), perceived severity (OR = 1.56; 95% CI = 1.43–1.70), perceived worry (OR = 2.03; 95% CI = 1.88–2.19), perceived likelihood of infection (OR = 1.26; 95% CI = 1.18–1.35), and perceived threat to physical health (OR = 1.52; 95% CI = 1.43–1.62) were all positively associated with perceived effectiveness and as each of those measures increased, odds of being in the higher perceived effectiveness group increased.

**Table 2:** Logistic regression analyses for the odds of being in the higher perceived-effectiveness group.

Variable	Unadjusted Analysis OR (95% CI)	Adjusted Analysis aOR (95% CI)	Best Fit Model aOR (95% CI) (p<0.01)^a^
*Demographic Characteristics*			
Age	**1.019 (1.015–1.023)**	**1.01 (1.00–1.02)**	**1.01 (1.00–1.01)**
Sex			
*Male*	Ref	Ref	Ref
*Female*	**1.33 (1.16–1.52)**	**1.33 (1.11–1.60)**	**1.34 (1.13–1.59)**
Education			
*Less than HS grad*, *HS grad*, *GED*	Ref	Ref	n/a
*Some college/Associate’s degree*	1.37 (1.14–1.66)	1.15 (0.90–1.47)	
*Bachelor’s degree*	1.33 (1.10–1.60)	0.90 (0.69–1.17)	
*Graduate school*	1.42 (1.15–1.74)	0.95 (0.70–1.28)	
Work in healthcare			n/a
*Yes*, *currently*	Ref	Ref	
*Yes*, *in the past*	1.28 (1.00–1.64)	1.10 (0.77–1.57)	
*No*, *never*	1.72 (1.42–2.09)	1.31 (0.97–1.76)	
Currently employed (earning income)			
*Yes*, *full time (35+ hours per week)*	Ref	Ref	n/a
*Yes*, *part time*	**0.78 (0.63–0.96)**	1.06 (0.80–1.40)	
*Yes*, *furloughed with pay*	**0.57 (0.37–0.88)**	0.78 (0.43–1.42)	
*Yes*, *furloughed without pay*	1.05 (0.77–1.42)	0.93 (0.63–1.39)	
*No*, *looking for work*	1.15 (0.91–1.46)	**1.78 (1.27–2.50)**	
*No*, *not looking for work*	1.16 (0.98–1.37)	0.94 (0.73–1.21)	
*Other*	1.42 (0.72–2.81)	0.98 (0.39–2.45)	
Household income (2019)			
*Less than $25*,*000*	Ref	Ref	n/a
*$25*,*000-$74*,*999*	**1.28 (1.08–1.53)**	**1.33 (1.06–1.68)**	
*$75*,*000-$149*,*999*	**1.35 (1.13–1.61)**	**1.31 (1.01–1.70)**	
*$150*,*000 or more*	**1.35 (1.06–1.71)**	1.20 (0.85–1.70)	
Political views			
*Liberal*	Ref	Ref	Ref
*Moderate*	**0.75 (0.63–0.88)**	**0.78 (0.63–0.96)**	**0.79 (0.64–0.97)**
*Conservative*	**0.57 (0.47–0.68)**	**0.61 (0.49–0.77)**	**0.62 (0.49–0.78)**
*Healthcare Characteristics*			
Pre-existing condition that would make COVID-19 more severe			n/a
*Yes*	Ref	Ref	
*No*	**0.74 (0.64–0.85)**	1.02 (0.82–1.26)	
Ever received a COVID-19 test			
*Yes*	Ref	Ref	n/a
*No*	**1.65 (1.33–2.04)**	1.22 (0.85–1.77)	
Know anyone who has had COVID-19			
*Yes*, *they had positive test*	Ref	Ref	Ref
*Believe so but unable to get tested*	**0.44 (0.34–0.57)**	**0.54 (0.38–0.77)**	**0.53 (0.37–0.74)**
*No*, *didn’t know anyone with COVID-19*	**0.74 (0.63–0.87)**	0.87 (0.70–1.08)	0.88 (0.71–1.09)
*Health Belief Variables*			
High Commitment Altruism	**1.12 (1.04–1.20)**	0.98 (0.87–1.11)	n/a
Low Commitment Altruism	**1.67 (1.54–1.80)**	**1.44 (1.26–1.64)**	**1.41 (1.27–1.56)**
Mean perceived severity of COVID-19 (4-items, range:1–5)	**1.56 (1.43–1.70)**	**0.85 (0.73–0.98)**	**0.84 (0.73–0.96)**
Mean COVID-19-related worry (3-items; range:1–5)	**2.03 (1.88–2.19)**	**1.74 (1.55–1.94)**	**1.82 (1.64–2.02)**
Threat of COVID-19 infection (individual items, of those not previously infected)			
*Likelihood of infection (1 = not at all; 5 = extremely)*	**1.26 (1.18–1.35)**	**0.85 (0.77–0.94)**	**0.85 (0.77–0.94)**
*Threat to physical health (1 = not at all; 5 = extremely)*	**1.52 (1.43–1.62)**	**1.33 (1.20–1.47)**	**1.32 (1.20–1.45)**
Believe COVID-19 is major problem in community			
*Yes*	Ref	Ref.	n/a
*No*	**0.49 (0.42–0.56)**	**0.78 (0.64–0.94)**	

^a^At 0.01 significance level, the variables were removed in the following order: pre-existing condition, hi commitment altruism, test to check for COVID-19, education, income, employed in healthcare field, believe COVID-19 is problem in community, and employment status.

### Adjusted regression models

For full regression results, see Table 2. In adjusted analyses, the following variables were not significant at p<0.01 and were removed from the model: having a health condition that would make COVID-19 more severe (p = 0.870), high commitment altruism (p = 0.767), having been tested for COVID-19 (p = 0.260), education (p = 0.190), income (p = 0.097), being employed in the healthcare field (p = 0.069), believing COVID-19 is a problem in their community (p = 0.018), and employment status (p = 0.022).

In the reduced model, the only demographic variables that remained were age, sex, and political views. Age was positively associated with perceived effectiveness and as age increased, odds of being in the higher perceived effectiveness group increased (aOR = 1.01; 95% CI = 1.00–1.01). Females also had higher perceived effectiveness of COVID-19 prevention measures (aOR = 1.34; 95% CI = 1.13–1.59). Perceived effectiveness of NPI was associated with political views and, compared to liberals, moderates (aOR = 0.79; 95% CI = 0.64–0.97) and conservatives (aOR = 0.62; 95%CI = 0.49–0.78) both had lower odds of being in the higher perceived effectiveness group. The only healthcare variable that remained in the model was whether someone knew anyone with COVID-19. Those who thought they knew someone with COVID-19, but said the person was unable to get tested, had lower odds of being in the higher perceived effectiveness group as compared to those who knew someone with a positive test (aOR = 0.53; 95% CI = 0.37–0.74).

Almost all of the health belief variables remained in the model, indicating a stronger association of health beliefs with perceived effectiveness than demographic characteristics or healthcare characteristics. However, some of the health belief variables switched directions in the adjusted model. Low commitment **altruism** (aOR = 1.41; 95% CI = 1.27–1.56), COVID-19-related worry (aOR = 1.82; 95% CI = 1.64–2.02), and perceived threat to physical health (aOR = 1.32; 95% CI = 1.20–1.45) all stayed consistent in their direction and were all positively associated with perceived effectiveness of COVID-19 prevention measures. On the other hand, perceived severity of COVID-19 (aOR = 0.84; 95% CI = 0.73–0.96) and perceived likelihood of infection (aOR = 0.85; 95% CI = 0.77–0.94) switched directions and were negatively associated with perceived effectiveness of COVID-19 prevention measures.

## Discussion

This is among the first studies to examine the public’s perception of the effectiveness of NPIs to prevent the spread of SARS-CoV-2. Despite recent public health messaging attempting to encourage these behaviors [5], there are media reports that only 65% of people wear masks in public stores and only 44% reported that most people in their communities are wearing masks [30]. Furthermore, there have been reports that wearing a mask (or not wearing a mask) has now become a political statement [31]. It is important to understand the public’s perceptions of the effectiveness of NPIs in preventing the spread of SARS-CoV-2. Given the overall high rates of perceived effectiveness of the six behaviors we assessed, as well as a recent CDC study that found widespread support for most NPI [32], it appears the issue is not that people have low perceived response efficacy of NPI to mitigate the spread of SARS-CoV-2. Rather, due to the strong effect of perceived worry on the rest of the variables in the model, the issue may be to emphasize the threat of the disease to the person’s physical health to highlight it is important to engage in these effective preventive health behaviors.

Given the strong association of perceived worry and the inverse association of perceived severity and likelihood of infection when accounting for worry, it appears there may be a strong influence of fatalistic beliefs for some individuals when it comes to preventing COVID-19 disease. People may believe that infection is inevitable and, once worry is taken into account, increases in perceived severity and likelihood of infection are actually associated with a decrease in perceived effectiveness. This has sometimes been seen in other severe diseases. For example, recent research has shown there are people who have fatalistic beliefs regarding the development of cancer and, as such, are less likely to engage in cancer preventive behaviors such as screening programs [33,34]. Messaging around COVID-19 prevention should focus on increasing perceived threat of disease, while reducing fatalistic beliefs by highlighting infection is not inevitable if a large enough proportion of the population engage in these prevention efforts.

In addition, it may be that working to increase engagement in these NPIs will need to involve a shift in public opinion and culture, particularly in the U.S. Recent reports have shown significantly less opposition to wearing masks in certain countries where mask-wearing was already common, including Japan, China, and South Korea [35]. In contrast, in the U.S., the culture is highly individualistic and resistance to wearing masks appears to be related to claims that individual liberty is valued over communal well-being [36,37]. Indeed, one report demonstrated men, in particular, were resistant to wearing masks due to the perception that it made them appear weak [38]. Our research did show men had lower perceptions of the effectiveness of these COVID-19 prevention measures compared to women. Furthermore, people with moderate and conservative political ideologies also had lower perception of NPI effectiveness. Groups with lower perceived effectiveness may be particularly important potential targets for interventions aimed at increasing adoption of NPIs to reduce the spread of SARS-CoV-2. Another variable that was associated with perceived effectiveness was low commitment altruism, which addressed behaviors like donating money or clothing to a charity. It may be that this scale tapped into a general sense of social responsibility, which would explain why higher scores predicted greater perceived effectiveness of NPI. Without effective messaging resulting in a shift in public opinion and greater adoption of NPIs, we may not be able to effectively mitigate the spread of the virus until a vaccine is developed and widely adopted.

The results should be interpreted in light of some limitations. First, these data are cross-sectional and causal relationships cannot be determined. Related, quantitative data only provide a limited look at this topic, so future work should consider the use of qualitative methods as well. Second, the data were collected at the beginning of May, 2020. While this is a rapid turnaround time between data collection and publication under normal circumstances, these are not normal circumstances. It is possible public perceptions of these behaviors have changed in the weeks since data were collected, especially given the recent moves by local governments to reopen public spaces and a resurgence of cases [39]. Third, data are self-report and are subject to recall and social desirability biases. However, given the anonymous nature of the survey, we believe social desirability bias has been appropriately reduced.

The COVID-19 pandemic is unprecedented in our lifetimes. With a novel virus, there is no population immunity, limited effective treatments, and no prophylactic vaccinations. While there have been strides made in the last several months regarding treatment, the safest possibility for each individual and for the public as a whole involves slowing the spread through non-pharmaceutical interventions. This study found people generally perceived as highly effective the wearing of masks, social distancing, washing hands, and covering mouths when coughing or sneezing. However, perceptions of effectiveness varied significantly on the basis of demographics, COVID-19-related experiences, and health beliefs. Health communication should therefore focus on the importance of NPI in protecting oneself as well as social contacts and work to depoliticize perceptions around wearing masks and social distancing.

## Supporting information

S1 Appendix Perceived effectiveness survey items. (DOCX)

S1 File (SAV)
