# Supplementary material for: Public perceptions of the effectiveness of recommended non-pharmaceutical intervention behaviors to mitigate the spread of SARS-CoV-2
Source: PLoS One. 2020 Nov 4;15(11):e0241662. doi: 10.1371/journal.pone.0241662 (PMC7641367; doi:10.1371/journal.pone.0241662)
Supplement: S1 Appendix — (DOCX) [file pone.0241662.s001.docx]

S1 Appendix. Perceived Effectiveness Survey Items

| In this set of questions, think about your day-to-day experiences during the COVID-19 pandemic.  In order to prevent catching COVID-19, the public has been advised to do certain behaviors.  For each item below, please indicate **how effective you think each behavior is to protect *yourself from being infected with COVID-19.*** | | | | | |
| --- | --- | --- | --- | --- | --- |
|  | Not effective at all | A little effective | Somewhat effective | Very effective | Extremely effective |
| Practicing social distancing by leaving at least six feet between you and other people (this does not include people you live with) |  |  |  |  |  |
| Frequently washing your hands for 20 seconds with warm water and soap |  |  |  |  |  |
| Avoiding touching your face |  |  |  |  |  |
| In order to prevent spreading COVID-19 to other people, the public has been advised to do certain behaviors.  For each item below, please indicate **how effective you think each behavior is to prevent you from *spreading COVID-19 to other people*.** | | | | | |
|  | Not effective at all | A little effective | Somewhat effective | Very effective | Extremely effective |
| Wearing a mask anytime you leave the house to go out in public |  |  |  |  |  |
| Practicing social distancing by leaving at least six feet between you and other people (this does not include people you live with) |  |  |  |  |  |
| Covering your mouth when you cough |  |  |  |  |  |
